# Development and Cross-Validation of a Predictive Equation for Fat-Free Mass in Brazilian Adolescents by Bioelectrical Impedance

**DOI:** 10.3389/fnut.2022.820736

**Published:** 2022-03-17

**Authors:** Roberto Fernandes da Costa, Analiza M. Silva, Kalina Veruska da Silva Bezerra Masset, Tatianny de Macêdo Cesário, Breno Guilherme de Araújo Tinoco Cabral, Gerson Ferrari, Paulo Moreira Silva Dantas

**Affiliations:** ^1^Physical Education Department, Health Sciences Center, Federal University of Rio Grande do Norte, Natal, Brazil; ^2^Exercise and Health Laboratory, CIPER, Faculdade Motricidade Humana, Universidade de Lisboa, Lisbon, Portugal; ^3^Escuela de Ciencias de la Actividad Física, el Deporte y la Salud, Universidad de Santiago de Chile (USACH), Santiago, Chile; ^4^Grupo de Estudio en Educación, Laboratorio de Rendimiento Humano, Actividad Física y Salud (GEEAFyS), Universidad Católica del Maule, Talca, Chile

**Keywords:** body composition, bioelectrical impedance analysis (BIA), fat-free mass (FFM), fat mass, equations, mathematical models, cross-validation

## Abstract

The bioelectrical impedance analysis (BIA) is one of the most commonly used techniques for assessing body composition in a clinical setting and in field approaches, as it has the advantages of easy application, fast, and non-invasive, in addition to its relatively low cost. However, the available predictive equations need to be valid for the evaluated subjects. The aim of this study was to verify the validity of several published BIA equations in estimating fat-free mass (FFM) among Brazilian adolescents, in addition to developing and cross-validating a BIA equation to estimate FFM appropriate for Brazilian adolescents. This is a cross-sectional study with 257 adolescents (128 girls) aged 10–19 years, randomly divided into two groups, namely, development (*n* = 172) and cross-validation (*n* = 85). The standard technique for assessing FFM was dual X-ray absorptiometry (DXA). The paired *t*-test, multiple regression, and the Bland-Altman plots were used to test the validity of the proposed models and to perform cross-validation of the model. The equation derived in this study was as follows: FFM = −17.189 + 0.498 (Height^2^/Resistance) + 0.226 Weight + 0.071 Reactance − 2.378 Sex + 0.097 Height + 0.222 Age; *r*^2^ = 0.92; standard error of the estimate = 2.49 kg; the new equation for FFM showed better agreement when compared with that of the equations developed in other countries. In conclusion, the newly developed equations provide a valid FFM estimation and are recommended for Brazilian adolescents with similar characteristics.

## Introduction

Body composition is an important component of the health-related physical fitness of children and adolescents (1, 2) and, therefore, deserves prominence in the prescription and monitoring of dietary and physical exercise programs. In addition, it is fundamental for the identification and monitoring of nutritional deviations (3–5).

The World Health Organization (WHO) defines adolescents as young people aged 10–19 years (6). The growth and development processes that occur in adolescence cause profound changes in the quantities and distribution of the different body components and, hence, the need to monitor these changes for the assessment of health status (4, 7). Furthermore, body composition variables in adolescents are inherently challenging because of the rapid growth-related changes in height, weight, fat-free mass (FFM), and fat mass (FM), but they are essential for the quality of the clinical follow-up (4).

Several techniques have been used to assess the body composition of children and adolescents, and dual X-ray absorptiometry (DXA) is one of the standard reference methods and non-invasive measurements for FFM and FM (8–10). However, its use requires high-cost equipment and specialized technical personnel, which makes it unfeasible in clinical and field situations (11). Although DXA cannot be considered the gold standard for the determination of FFM at the molecular level, and the four-compartment model (4C) is the most suitable reference method to assess FM and FFM at the molecular level (12), due to the complexity of the technique (13), the use of DXA to derive BIA equations has been widely accepted (14, 15). In addition, in Brazil, two previous studies have developed equations to estimate the FFM of men (16) and women (17) aged 20–59 years, with high validity, using DXA as a reference technique.

Whole-body-based techniques, such as anthropometry and bioelectrical impedance analysis (BIA), used as predictors in regression equations, developed and validated using DXA as the reference method, are a viable alternative for clinical evaluation since they are non-invasive, ideal for quantitative estimates of FFM and FM based on mathematical models, portable, and relatively low cost (9, 18, 19). However, it is essential that predictive equations are selected for subjects with similar characteristics regarding gender, age group, pubertal stage, ethnicity, and nutritional status (20, 21). These characteristics make BIA the most used tool to assess body composition worldwide (22).

In Brazil, no studies have developed predictive equations of FFM by bioelectrical impedance for adolescents using DXA as a reference method, and equations developed in other countries (15, 23, 24) are frequently used, which may limit the validity of the results obtained.

Thus, the objectives of this study were: (a) to verify the validity of several BIA equations, published in different countries, in order to estimate FFM in Brazilian adolescents, and (b) to develop and cross-validate BIA equations to estimate FFM in Brazilian adolescents using DXA as a reference method for assessing body composition.

## Materials and Methods

This is an observational study with a cross-sectional design for the development and cross-validation of a regression equation to estimate body composition, carried out between January 2018 and April 2019, in Natal, which has an estimated population of 884,122 inhabitants according to the IBGE (Brazilian Institute of Geography and Statistics) (25).

### Sample

**FIGURE 1:**
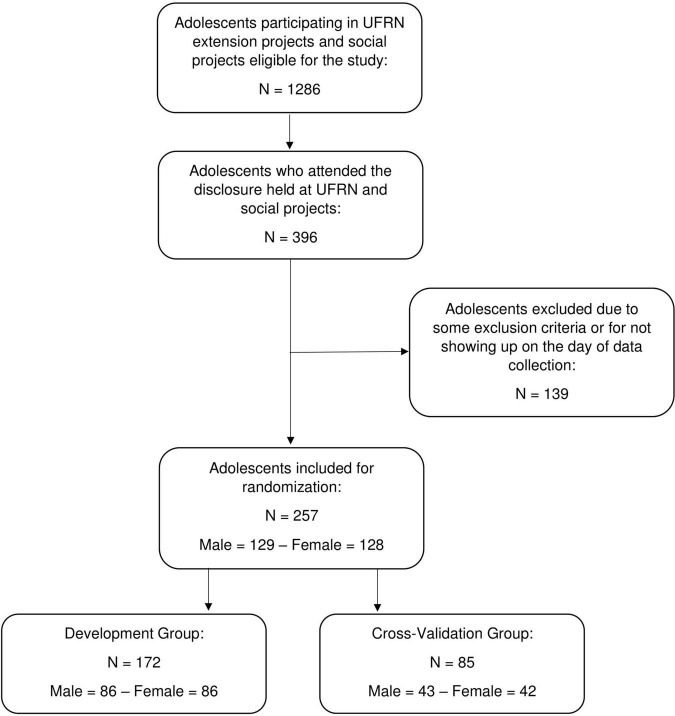
Flowchart of the study protocol.

The convenience sample consisted of 257 adolescents (128 girls), aged 10–19 years, from the northeast region of Brazil, who were recruited through dissemination among the participants of university extension projects from the Physical Education Department of the Federal University of Rio Grande do Norte (UFRN) and two social projects maintained by the federal government (Figure 1). After their inclusion in the study, the sample was randomly divided into two groups, namely, the development of a predictive equation for FFM (*n* = 172) and cross-validation (*n* = 85). For the sample size calculation, using FFM as a primary outcome, we considered a medium to small effect size (0.12) with five predictors (independent variables), with a type I error of 5% and a power of 95%. Using these parameters, a total of 171 participants were required.

The inclusion criteria were non-athlete adolescents of both sexes aged 10–19 years, regardless of nutritional status, without any medical condition that could interfere with body composition results. The exclusion criteria were pregnancy; hypovolemic or hypervolemic conditions, including diet, diuretic, or corticoid use; edema; individuals with any physical disability or chronic disease; or individuals who had a prosthesis that could alter the results of the body composition assessment. It should be noted that the option for non-athletes is based on possible differences in body composition found in athletes, which can be defined as people in competitive sporting events individually or in engaged teams, with high physical performance and specific training methods (26, 27). Although there are people who, despite not being athletes, may present body composition characteristics similar to those of athletes (28), they could be included in the study because they are conceptually recognized as non-athletes.

All data collections were conducted in a single visit by each participant to the laboratory to perform, sequentially, anthropometric measurements, BIA and DXA assessments, in addition to assessing the pubertal stage. All participants and their parents or legal guardians were informed about the study protocol and signed a free and informed assent/consent form (FICF). The overarching protocol was approved by the Research Ethics Committee of the University Hospital Onofre Lopes—HUOL/UFRN (#34804414.7.0000.5292).

### Anthropometric Measurements

Anthropometric measurements were performed by one physical education professional, who was properly trained in accordance with international recommendations (29). Weight was measured using a digital scale with 0.1 kg resolution from Sanny^®^, model BL200PP (American Medical do Brasil, São Bernardo do Campo, Brazil), with the participants being barefoot and wearing light clothes. In addition, all jewelry and metals were removed for this and all subsequent measurements. Height was measured using a stadiometer from Sanny^®^ with a resolution of 0.1 cm, Caprice model (American Medical do Brasil, São Bernardo do Campo, Brazil), with the participants being barefoot and in orthostatic position. Body mass index (BMI) was calculated by dividing body mass (kg) by the square of height (m), and adolescents were classified as underweight, normal weight, overweight, or obese using growth charts proposed by the WHO (30).

### Bioelectrical Impedance Analysis

The assessment by BIA, for the determination of resistance (R), reactance (Xc), and phase angle (PhA), was conducted with single-frequency tetrapolar equipment (50 kHz) at a current of 800 μA, in equipment from Sanny^®^, BIA1010 model (American Medical do Brasil, São Bernardo do Campo, Brazil). The BIA equipment validity measurement was periodically measured with an electrical resistor and capacitor. Calibration values were considered normal if the R was not higher than 500 ± 5 ohm (Ω) and Xc was not higher than 52 ± 0.5 Ω, according to the manufacturer’s instructions. The Sanny bioimpedance equipment was chosen because it is the only one manufactured in Brazil, which implies easy access in the country and relatively low operating cost.

To verify the quality of the measurements obtained by the equipment, reproducibility was calculated in a previous study with 46 women from the northeast region of Brazil. The results obtained were a coefficient of variation (CV) of 0.17 and 0.72% for R and Xc, respectively, and a technical error of measurement (TEM) of 0.76 Ω (0.22%) and 0.35 Ω (0.92%) for R and Xc, respectively (17).

Participants were evaluated after lying down for 10 min in the supine position on a non-conductive stretcher. Arms and legs were abducted 30° from the midline of the body. To avoid short-circuiting in obese participants, a foam device was used between the lower limbs. The skin was cleaned with 70% alcohol before placing the electrodes, which were positioned on the dorsal surface of the wrist, hand, ankle, and foot, in the right hemibody. The evaluated individuals were asked to fast for at least 4 h before the assessment, not to perform any strenuous physical exercise in the previous 24 h, and not to consume alcohol in the previous 48 h. In addition, they were asked to empty their bladder 30 min before the assessment. The resistance index (Ht^2^/R) was calculated by dividing the square of height (m) by R (Ω).

### Dual X-Ray Absorptiometry

Dual X-ray absorptiometry was performed with Lunar Prodigy equipment, NRL 41990 model (GE Lunar, Madison, WI, United States), by a laboratory technician experienced in radiological evaluation. The scan was conducted with the participants lying in the supine position along the longitudinal axis of the midline of the table. Feet were positioned together and stuck at the level of the fingers to immobilize the legs, while the hands were held in the prone position within the scanning region of the equipment. The participants remained still during the digitalization process. Measurements were performed following the recommendations proposed by Nanna et al. (31). Body composition was determined using the enCoreTM 2011 version 13.6 software (GE Health Lunar). As described elsewhere, CV for FM, bone mineral content (BMC), and lean soft tissue (LST) using the current equipment were 0.74, 0.28, and 0.26%, respectively. TEM were 0.25, 0.02, and 0.25 kg to FM, BMC, and LST, respectively (32). The FFM was obtained by the sum of BMC and LST (FFM = BMC + LST).

### Pubertal Stage

For the identification of the pubertal stage, the self-assessment technique (33) was used, based on the classification proposed by Tanner (34), which uses five levels to classify the development of the breasts (i.e., M1, M2, M3, M4, and M5) for girls and the development of the genitalia (i.e., G1, G2, G3, G4, and G5) for boys, with them being considered prepubescent adolescents than those who report being in M1 and G1, pubescents from M2 to M4 or G2 to G4, and postpubescent M5 and G5. After the anthropometric assessment, the adolescents were taken individually to a room where the researcher explained the importance of this assessment and presented boards with images of breasts/genitalia and pubic hair. This procedure was carried out with great professionalism and rigor to avoid causing embarrassment or discomfort to the adolescents, as well as any inappropriate representation on the boards. For the data analysis, we chose to use organ development for both sexes, since pubic hair alone can be influenced by ethnic characteristics, as previously described (35).

### Statistical Analysis

The Kolmogorov-Smirnov test was applied to verify the normal distribution of data. The descriptive analysis consisted of mean and standard deviation of all study variables, and the comparisons between groups were performed by Student’s *t*-test for independent samples. The stepwise multiple regression analysis was used to propose the predictive equation for FFM. The stepwise regression analysis was conducted using FFM obtained by DXA as a dependent variable and age, weight, height, BMI, R, Xc, PhA, R index, and pubertal stage as possible independent variables. During model development, normality of residuals and homogeneity of variance were tested. Significance at *p* < 0.05 was established as the criterion for inclusion of a predictor, whereas removal criteria were set at *p* > 0.1. If more than one variable remained in the model, and to assess multicollinearity, a variance inflation factor (VIF) and the tolerance (reciprocal of VIF) were calculated for each independent variable, and a VIF < 10 or tolerance higher than 0.1 was considered appropriate (36, 37). To verify the validity of the proposed equation, the estimated mean results were compared with the mean results measured in DXA by the paired *t*-test. In addition, the Pearson’s correlation coefficient (r), coefficient of determination (r^2^), and standard error of the estimate (SEE) were calculated.

The approach proposed by Lin (38) was used for the concordance correlation coefficient (CCC) analysis to verify the validity (Cb) and accuracy (ρ) between estimated and measured FFM values. For the cross-validation of the equation proposed in this study, a multiple regression analysis was performed.

In turn, the new BIA equation accuracy was evaluated using pure error (PE), which was calculated as the squared root of the mean of the sum of squared differences between the measurement and estimate of FFM (15). The Bland-Altman (39) plots were used to verify bias and concordance between FFM measurement and estimate, in which the limits of agreement (LOAs) were defined as the mean of differences ± 1.96 standard deviations, including the analysis of the correlation between the mean and the difference of the methods. Additionally, the same procedures were used to test the validity of the other eight equations proposed for estimating FFM in adolescents (15, 23, 24, 40–44). Analyses were carried out with the statistical package SPSS v.20.0 (SPSS Inc., IBM Corp., Armonk, New York, NY, United States) and MedCalc version 12.5.0. Statistical significance of *p* < 0.05 was considered for all tests.

## Results

Table 1 presents the physical characteristics and body composition variables for the developmental and cross-validation groups, as well as for the whole sample with no differences observed between the two groups (i.e., developmental and cross-validation) (*p* > 0.05). The characteristics of the samples from the eight equations tested in this study are shown in Table 2.

**TABLE 1:** Descriptive [mean ± sd or *n* (%)] characteristics and body composition of development and cross-validation groups.

	Development group (DG)			Cross-validation group (CVG)		
	Male (*n* = 86)	Female (*n* = 86)	Total sample (*n* = 172)	Male (*n* = 43)	Female (*n* = 42)	Total sample (*n* = 85)
Age (years)	13.6 ± 2.9	14.5 ± 3.6	14.1 ± 3.3	13.7 ± 3.1	14.2 ± 3.1	14.0 ± 3.1
**Pubertal stage - *n* (%)**						
Prepubertal	11 (12.8)	7 (8.1)	18 (10.5)	8 (18.6)	2 (4.8)	10 (11.8)
Pubescent	52 (60.5)	48 (55.8)	100 (58.1)	23 (53.5)	29 (69.0)	52 (61.2)
Postpubertal	23 (26.7)	31 (36.0)	54 (31.4)	12 (27.9)	11 (26.2)	23 (27.1)
**BMI status (30) - *n* (%)**						
Low weight	8 (9.3)	4 (4.7)	12 (7.0)	1 (2.3)	5 (11.9)	6 (7.1)
Normal weight	61 (70.9)	58 (67.4)	119 (69.2)	32 (74.4)	27 (64.3)	59 (69.4)
Overweight	15 (17.4)	18 (20.9)	33 (19.2)	9 (20.9)	6 (14.3)	15 (17.6)
Obesity	2 (2.3)	6 (7.0)	8 (4.7)	1 (2.3)	4 (9.5)	5 (5.9)
Weight (kg)	47.7 ± 16.5	50.9 ± 15.1	49.3 ± 15.9	49.0 ± 16.0	48.1 ± 12.4	48.6 ± 14.3
Height (cm)	156.4 ± 14.1	157.2 ± 10.8	156.8 ± 12.5	157.1 ± 14.9	156.1 ± 9.0	156.6 ± 12.3
BMI (kg/m^2^)	18.9 ± 3.8	20.2 ± 4.2	19.6 ± 4.1	19.3 ± 3.6	19.7 ± 4.8	19.5 ± 4.2
FM (kg)	10.9 ± 6.0	17.3 ± 7.7	14.1 ± 7.6	11.5 ± 5.4	16.5 ± 6.9	14.0 ± 6.6
FM (%)	22.7 ± 7.2	32.9 ± 7.0	27.8 ± 8.9	23.7 ± 8.1	33.4 ± 7.0	28.5 ± 9.0
FFM (kg)	36.7 ± 12.8	33.7 ± 8.8	35.2 ± 11.1	37.3 ± 13.4	31.6 ± 7.0	34.6 ± 11.1
Resitance (Ω)	634.1 ± 112.7	684.8 ± 101.5	659.4 ± 109.9	634.7 ± 119.4	714.0 ± 108.6	673.9 ± 120.3
Reactance (Ω)	63.6 ± 10.6	65.9 ± 10.3	64.8 ± 10.5	67.1 ± 12.6	67.7 ± 9.6	67.2 ± 11.2
Phase angle (°)	5.8 ± 1.0	5.6 ± 0.9	5.7 ± 0.9	6.2 ± 1.5	5.5 ± 1.0	5.8 ± 1.3
Resistance Index (Ht^2^/R)	41.2 ± 14.4	37.4 ± 9.2	39.3 ± 12.2	41.7 ± 14.7	35.1 ± 7.3	38.5 ± 12.1

*BMI, body mass index; FM, fat mass; FFM, fat-free mass.*

**TABLE 2:** Sample characteristics of the bioelectrical impedance analysis (BIA) equations for the prediction of fat-free mass (FFM) in adolescents.

	Age (years)	*n* M/F	Ethnicity	Reference methods
Deurenberg et al. (23)	7–25	130 M, 116 F	C	UW
Houtkooper et al. (24)	10–19	225 M/F	C	UW and DD
Sun et al. (15)	12–94	669 M, 944 F	C and As	DD, UW, and DXA
Boileau (40)	8–16	NR	C	UW and DD
Horlick et al. (41)	4–18	645 M, 602 F	C, AA, Af, and As	DD and DXA
Schaefer et al. (42)	3–19	59 M, 53 F	C	TBK
Suprasongsin et al. (43)	10–22	21 M, 21 F	C	ID
Wang et al. (44)	9–19	127 M, 128 F	As	DXA

*M, male; F, female; NR, no report; C, Caucasian; As, Asian; AA, American African; Af, African; UW, underwater weighing; DD, deuterium dilution; DXA, dual-energy X-ray absorptiometry; TBK, total body potassium; ID, isotope dilution, heavy water tracer.*

**FIGURE 2:**
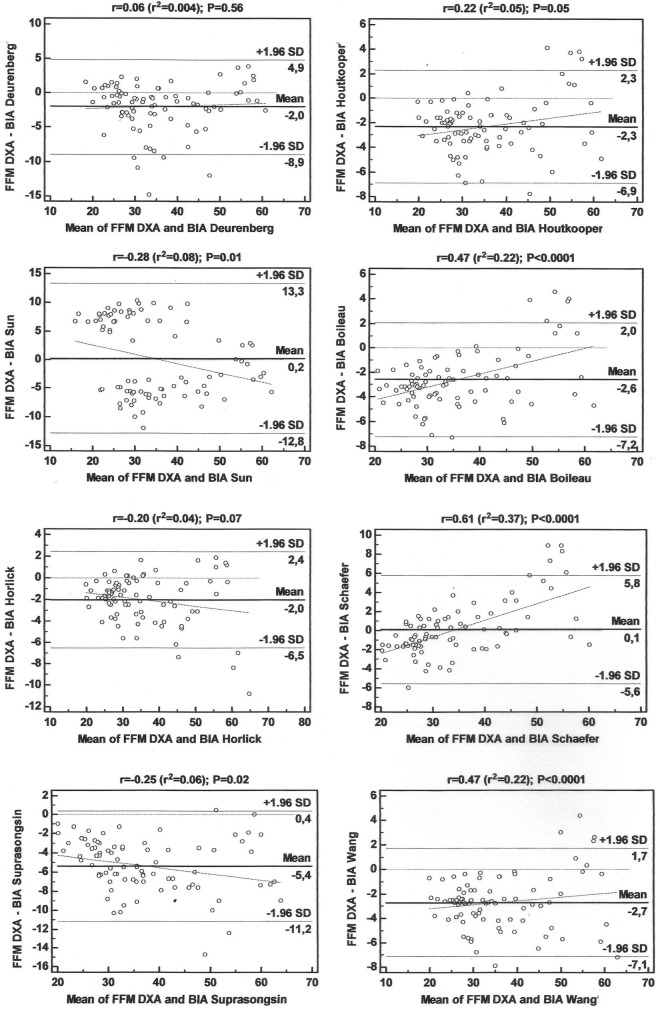
Bland-Altman plots for the concordance limits between values determined by the reference method (DXA) and eight equations for fat-free mass (FFM) in adolescents.

The analysis of the validity of the eight equations (Table 3), developed in other countries, showed that, only for three equations (23, 41, 44), no association was found between the mean and the difference of the BIA and DXA methods (*p* > 0.05). The mean difference in the Bland-Altman plot was not different from zero in just two equations (15, 42; *p* > 0.05). All equations showed high LOA, indicating poor agreement with the reference method (Figure 2). These results justified the need to develop and validate a specific equation for our population.

**TABLE 3:** Cross-validation of fat-free mass predictive new equation, and validation of other published equations.

			CCC analysis				
	FFM (kg)	*p*-value^*^	CCC	ρ	C_*b*_	r^2^	PE (kg)
New equation	34.8 ± 10.6	0.322	0.984	0.985	0.999	0.97	1.95
Deurenberg et al. (23)	36.6 ± 10.9	<0.001	0.932	0.949	0.983	0.90	4.05
Houtkooper et al. (24)	36.9 ± 10.6	<0.001	0.955	0.978	0.977	0.96	3.27
Sun et al. (15)	34.3 ± 12.9	0.732	0.847	0.857	0.988	0.73	6.63
Boileau (40)	37.2 ± 10.0	<0.001	0.946	0.980	0.965	0.96	3.50
Horlick et al. (41)	36.6 ± 11.6	<0.001	0.964	0.981	0.983	0.96	3.05
Schaefer et al. (42)	34.5 ± 9.3	0.715	0.960	0.974	0.985	0.95	2.88
Suprasongsin et al. (43)	40.5 ± 11.8	<0.001	0.870	0.969	0.898	0.94	6.13
Wang et al. (44)	37.3 ± 10.8	<0.001	0.949	0.979	0.969	0.96	3.51

FFM, fat-free mass; CCC, concordance correlation coefficient; ρ, accuracy; C_b_, validity; PE, pure error. *Differences between predictive equations and reference method by paired t test.

In preliminary analyses, we found no significant interaction with sex for any of the main independent predictor, and, thus, girls and boys were combined for the development of the prediction models.

Table 4 shows the regression model for the prediction of FFM (kg). A preliminary model was developed to estimate FFM, including anthropometric and BIA variables, that is, age, weight (W), height (H), BMI, R, Xc, PhA, resistance index (Ht^2^/R), and pubertal stage. Only variables contributing to the estimates using a backward stepwise approach were used in the model. The performance of the developed model can be observed by high coefficients of determination (r^2^ = 0.95) and low SEE (SEE = 2.5 kg).

**TABLE 4:** Regression model for the prediction of fat-free mass (kg).

Variables included in the model	Regression coefficient	*r* ^2^	SEE	*p*-value	Collinearity statistics	
					Tolerance	VIF
Constant	−17.189			<0.001		
Ht^2^/R	+0.498	0.916^a^	3.214	<0.001	0.144	6.961
Weight	+0.226	0.935^b^	2.850	<0.001	0.175	5.713
Reactance	+0.071	0.942^c^	2.689	<0.001	0.639	1.565
Sex	−2.378	0.947^d^	2.579	<0.001	0.693	1.443
Height	+0.097	0.949^e^	2.528	0.002	0.533	1.625
Age	+0.222	0.951^f^	2.498	0.027	0.355	2.817

*SEE, standard error of the estimate; VIF, variance inflation factor. Predictors: ^a^(Constant), Ht^2^/R. ^b^(Constant),s Ht^2^/R, weight. ^c^(Constant), Ht^2^/R, weight, and reactance; ^d^(Constant), Ht^2^/R, weight, reactance, and sex. ^e^(Constant), Ht^2^/R, weight, reactance, sex, and height; ^f^(Constant), Ht^2^/R, weight, reactance, sex, and age. The r^2^ change was significant for a, b, c, d, e, and, f.*

The resulting prediction model included is presented below:


$$\begin{array}{c} \mathrm{FFM}=-17.189+0.498\,\left({\mathrm{Height}}^{2}/\mathrm{Resistance}\right)+0.226\,\mathrm{Weight}+\\ 0.071\,\mathrm{Reactance}-2.378\,\mathrm{Sex}+0.097\,\mathrm{Height}+0.222\,\mathrm{Age} \end{array}$$

Sex:male= 0;female= 1

Estimated FFM by the specific equation developed in this study did not present significant differences in comparison with the value determined by DXA for both the development and cross-validation groups. All parameters used for proposing and validating the equation confirmed their validity. Additionally, no association was found between the mean and the difference of the methods (*r* = 0.113; *p* = 0.141).

The performance of the cross-validation of the new equation and the validity of the eight equations developed for adolescents in other countries are shown in Table 2, and the LOA for each of the equations is shown in Figure 2.

**FIGURE 3:**
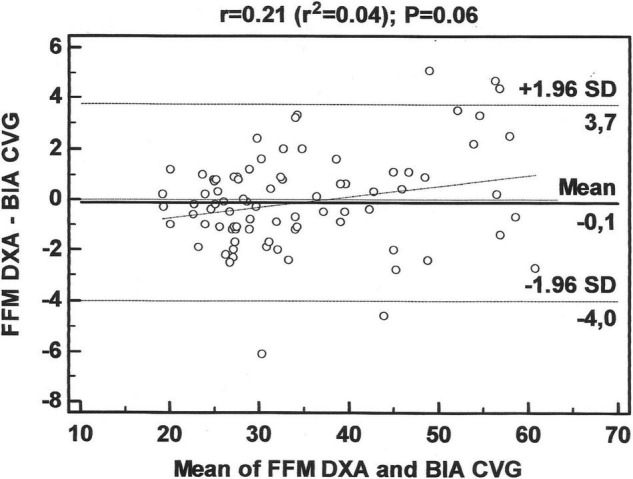
Bland-Altman plots for the concordance limits between values determined by the reference method (DXA) and the equation for fat-free mass (FFM) in adolescents, derived in this study.

Figure 3 presents the LOA for FFM between the standard method (DXA) and the BIA equation derived in this study. The mean difference in the Bland-Altman plot was not different from zero in the cross-validation group (*p* = 0.322). The LOA ranged between −4.0 and 3.7 kg to the cross-validation group, indicating a good agreement between the developed equation and the reference method.

## Discussion

The BIA is one of the most convenient techniques for assessing body composition in a clinical setting and in epidemiological approaches, mainly, because it is easy to apply, fast, and non-invasive, in addition to having a relatively low cost (5, 45, 46). The use of predictive equations that have been developed and cross-validated in groups with similar characteristics to those of the subjects that we intend to evaluate can reduce discrepancies among studies (7, 20, 47). Observing the lack of predictive equations for FFM in Brazilian adolescents, developed using DXA as a reference technique, this study aimed at developing and cross-validating an equation in a sample of Brazilian adolescents of both sexes, in addition to testing the validity of equations developed in other countries, used frequently in our country.

In this study, the most relevant predictor was the resistance index (Ht^2^/R), which explained alone 92% of the variability of our equation. The electrical properties of the human body may explain the use of BIA to estimate FFM by the resistance index. R of the conductor is expressed by R = ρL^2^/V, so V = ρL^2^/R, wherein ρ is the conductor resistivity, L is the length, and V is volume (48, 49). Thus, as the lean body mass contains a large amount of water, it presents low R to the flow of electric current, while the FM has greater R to the passage of current. Therefore, the R associated with height can be a good estimator of these body compartments.

The other variables that entered the model were weight, Xc, sex, and age. Considering that adolescence is a phase of profound morphological changes, which include body composition (4, 50, 51), and that the age at which each stage of puberty occurs can vary considerably (52), we listed the pubertal stage among the possible independent variables; however, this variable was not included in the model by the stepwise regression. The same was verified in the other studies that proposed predictive equations of FFM by BIA in adolescents, which we tested for validity in the sample of this study (15, 23, 24, 40–44).

In the sample of this study, mean BMI in boys (18.9 kg/m^2^) and girls (20.2 kg/m^2^) was similar to the mean BMI of a representative sample of 12- to 17-year-old boys (21.0 kg/m^2^) and girls (21.3 kg/m^2^) from the Brazilian cities with more than one hundred thousand inhabitants (53). Thus, although the sample was not representative of adolescents across Brazil, the relative body size of these adolescents seems to resemble that of other adolescents of the country living in larger cities. However, it is important that the techniques for body composition assessment that are used have been validated for the target population (37, 54, 55).

Although the use of predictive equations in subjects with different characteristics from those presented by the group of origin of the equations is questionable (15), we did not always have mathematical models validated for similar groups to the ones we wanted to evaluate. In addition, many BIA devices do not refer to the predictive equations available in their software (17). Thus, it is possible that health professionals are using inadequate equations for their patients, which indicates the need for studies to validate predictive equations that already exist in different population groups, as well as the development of new equations.

The equations developed for adolescents from other countries, tested in this study, did not prove to be valid for our sample. When comparing the predicted mean FFM values with those obtained by the reference technique, only two equations showed no significant difference (15, 42). However, all equations, including these two (15, 42), showed high limits of agreement, limiting their use at the individual level, for the subjects of this study.

A study carried out by Koury et al. (50) in Brazil, with a sample of 368 adolescent athletes aged 11–16 years, tested the validity of three equations (23, 41, 56), concluding that none of them was adequate for the evaluated sample. The authors developed gender-specific equations for the study sample, including skeletal maturity for boys and menarche status for girls as dependent variables, demonstrating good performance; however, there was no cross-validation of the new equations. We did not test the validity of these equations in our sample because we did not evaluate athletes and did not collect information about skeletal maturity.

A systematic review was carried out by Silva et al. (7) to identify predictive equations for assessing FM and FFM in healthy children and adolescents using the multicomponent molecular models as a reference method. Similar to this study, most of the equations were developed using DXA as a reference method, but a limited number of studies provided cross-validation results. The authors of the systematic review identified that, of the 33 equations analyzed, only seven were cross-validated, two studies examined the PE in the FFM estimate, and none of the studies examined the CCC, while the agreement between the methods was included only in three studies. Based on the limitations found in other studies, we sought to use the most recommended methodological practices for the development and cross-validation of predictive models of body composition assessment.

This study has several strengths. To our knowledge, this is the first study to develop and cross-validate a predictive equation of FFM by BIA, using DXA as a reference method, in Brazilian non-athlete adolescents. The equation developed in our study showed a high coefficient of determination and good limits of agreement in relation to the reference method, and all parameters used for the proposition and cross-validation of the model confirmed their validity for the studied population (15, 37–39, 57), which can be used to monitor changes in FFM resulting from growth, dietary programs, and physical exercises (14, 58, 59).

However, some limitations must also be addressed. This study included a sample of adolescents from only one region of the country, and ethnicity was not assessed. Other studies carried out in Brazil for the development of predictive equations by anthropometry (60) and BIA (32) also used ethnically mixed samples, miscegenation, and ethnic differences, which suggest the need to validate the equation proposed in the study in other regions of the country and with subjects of different ethnic origins. Another important issue concerns the standard technique used. The 4C model is the most appropriate reference method for assessing FM and FFM at the molecular level (12). However, due to the complexity of the technique (13), the use of DXA to derive BIA equations has been widely accepted (14, 15). Hydration status was not determined to ensure a euhydration state prior to body composition measures, although self-reported water intake was within the normal range, and pale morning urine was reported. It should be noted that the new equations are only useful for Brazilian adolescents with similar characteristics. In addition, further research should be conducted to test the accuracy of the new model in tracking FFM.

In conclusion, based on the results obtained, the equation developed in this study met the validation criteria to estimate FFM, while the equations developed in other countries were not considered valid for the studied sample. Thus, this new equation can be considered a good alternative for assessing the body composition of adolescents with similar characteristics by BIA due to the good validity presented.

## Data Availability Statement

The raw data supporting the conclusions of this article will be made available by the authors, without undue reservation.

## Ethics Statement

The studies involving human participants were reviewed and approved by Research Ethics Committee of the University Hospital Onofre Lopes – HUOL/UFRN (#34804414.7.0000.5292). Written informed consent to participate in this study was provided by the participants’ legal guardian/next of kin.

## Author Contributions

RC and KM: conceptualization. RC, KM, and TC: data curation. RC: formal analysis and writing—original draft. PD: funding acquisition and project administration. RC, KM, TC, BC, and PD: investigation. RC, AS, KM, BC, GF, and PD: writing—review and editing. All authors contributed to the article and approved the submitted version.

## Conflict of Interest

The authors declare that the research was conducted in the absence of any commercial or financial relationships that could be construed as a potential conflict of interest.

## Publisher’s Note

All claims expressed in this article are solely those of the authors and do not necessarily represent those of their affiliated organizations, or those of the publisher, the editors and the reviewers. Any product that may be evaluated in this article, or claim that may be made by its manufacturer, is not guaranteed or endorsed by the publisher.
