# Corrigendum: Development and cross-validation of a predictive equation for fat-free mass in Brazilian adolescents by bioelectrical impedance

**DOI:** 10.3389/fnut.2022.1128979

**Published:** 2023-01-13

**Authors:** Roberto Fernandes da Costa, Analiza M. Silva, Kalina Veruska da Silva Bezerra Masset, Tatianny de Macêdo Cesário, Breno Guilherme de Araújo Tinoco Cabral, Gerson Ferrari, Paulo Moreira Silva Dantas

**Affiliations:** ^1^Physical Education Department, Health Sciences Center, Federal University of Rio Grande do Norte, Natal, Brazil; ^2^Exercise and Health Laboratory, CIPER, Faculdade Motricidade Humana, Universidade de Lisboa, Lisbon, Portugal; ^3^Escuela de Ciencias de la Actividad Física, el Deporte y la Salud, Universidad de Santiago de Chile (USACH), Santiago, Chile; ^4^Grupo de Estudio en Educación, Laboratorio de Rendimiento Humano, Actividad Física y Salud (GEEAFyS), Universidad Católica del Maule, Talca, Chile

**Keywords:** body composition, bioelectrical impedance analysis (BIA), fat-free mass (FFM), fat mass, equations, mathematical models, cross-validation

In the original article, there was a mistake in Table 4 as published. The article included a table referring to another mathematical model, which does not refer to this study. The corrected Table 4 appears below.

**Table 4:** Regression model for the prediction of fat-free mass (kg).

**Variables included in the model**	**Regression coefficient**	***r* ^2^**	**SEE**	***p*-value**	**Collinearity statistics**	

					**Tolerance**	**VIF**
Constant	−17.189			< 0.001		
Ht^2^/R	+0.498	0.916^a^	3.214	< 0.001	0.144	6.961
Weight	+0.226	0.935^b^	2.850	< 0.001	0.175	5.713
Reactance	+ 0.071	0.942^c^	2.689	< 0.001	0.639	1.565
Sex	−2.378	0.947^d^	2.579	< 0.001	0.693	1.443
Height	+0.097	0.949^e^	2.528	0.002	0.533	1.625
Age	+0.222	0.951^f^	2.498	0.027	0.355	2.817

SEE, standard error of the estimate; VIF, variance inflation factor. Predictors:

a (Constant), Ht^2^/R.

b (Constant),s Ht^2^/R, weight.

c (Constant), Ht^2^/R, weight, and reactance;

d (Constant), Ht^2^/R, weight, reactance, and sex.

e (Constant), Ht^2^/R, weight, reactance, sex, and height;

f (Constant), Ht^2^/R, weight, reactance, sex, and age. The r^2^ change was significant for a, b, c, d, e, and, f.

In the original article, there was an error on page 5 in the section **Results**. In the presentation of the mathematical model for FFM estimation, the word “Fri” appears instead of the word “Sex.”

The corrected mathematical model included is presented below:


$$\begin{array}{l} \text{FFM}=-\;17.189+0.498\;({\text{Height}}^{2}\text{/Resistance})+0.226\text{ Weight }+0.071\text{ Reactance}\\ \;-\;2.378\text{ Sex}+0.097\text{ Height }+0.222\text{ Age} \end{array}$$

Sex: male = 0; female = 1

The authors apologize for this error and state that this does not change the scientific conclusions of the article in any way. The original article has been updated.

